# Transcriptome analysis of heat stress response in switchgrass (*Panicum virgatum* L.)

**DOI:** 10.1186/1471-2229-13-153

**Published:** 2013-10-06

**Authors:** Yong-Fang Li, Yixing Wang, Yuhong Tang, Vijaya Gopal Kakani, Ramamurthy Mahalingam

**Affiliations:** 1 Department of Biochemistry and Molecular Biology, Oklahoma State University, Stillwater, OK 74078, USA; 2 Samuel Roberts Noble Foundation, Genomics Core Facility, Ardmore, OK 73401, USA; 3 Department of Plant and Soil Sciences, Oklahoma State University, Stillwater, OK 74078, USA

**Keywords:** Switchgrass, Biofuel, Microarray, Heat stress, Transcriptome

## Abstract

**Background:**

Global warming predictions indicate that temperatures will increase by another 2-6°C by the end of this century. High temperature is a major abiotic stress limiting plant growth and productivity in many areas of the world. Switchgrass (*Panicum virgatum* L.) is a model herbaceous bioenergy crop, due to its rapid growth rate, reliable biomass yield, minimal requirements of water and nutrients, adaptability to grow on marginal lands and widespread distribution throughout North America. The effect of high temperature on switchgrass physiology, cell wall composition and biomass yields has been reported. However, there is void in the knowledge of the molecular responses to heat stress in switchgrass.

**Results:**

We conducted long-term heat stress treatment (38°/30°C, day/night, for 50 days) in the switchgrass cultivar Alamo. A significant decrease in the plant height and total biomass was evident in the heat stressed plants compared to controls. Total RNA from control and heat stress samples were used for transcriptome analysis with switchgrass Affymetrix genechips. Following normalization and pre-processing, 5365 probesets were identified as differentially expressed using a 2-fold cutoff. Of these, 2233 probesets (2000 switchgrass unigenes) were up-regulated, and 3132 probesets (2809 unigenes) were down-regulated. Differential expression of 42 randomly selected genes from this list was validated using RT-PCR. Rice orthologs were retrieved for 78.7% of the heat stress responsive switchgrass probesets. Gene ontology (GOs) enrichment analysis using AgriGO program showed that genes related to ATPase regulator, chaperone binding, and protein folding was significantly up-regulated. GOs associated with protein modification, transcription, phosphorus and nitrogen metabolic processes, were significantly down-regulated by heat stress.

**Conclusions:**

Plausible connections were identified between the identified GOs, physiological responses and heat response phenotype observed in switchgrass plants. Comparative transcriptome analysis in response to heat stress among four monocots – switchgrass, rice, wheat and maize identified 16 common genes, most of which were associated with protein refolding processes. These core genes will be valuable biomarkers for identifying heat sensitive plant germplasm since they are responsive to both short duration as well as chronic heat stress treatments, and are also expressed in different plant growth stages and tissue types.

## Background

Switchgrass (*Panicum virgatum* L.) is an economically important, warm-season, and widely adapted C4 perennial grass [1]. Due to its rapid growth rate, reliable biomass yield across locations, minimal requirements of water and nutrients, adaptability to growth on marginal lands and widespread distribution throughout North America, switchgrass has been selected in 1992 by the U. S. Department of Energy (USDOE) as a model herbaceous bioenergy crop for the development of renewable feed stock resource to produce transportation fuel [2]. Based on morphology and habitat preference, switchgrass has been classified into two groups: lowland ecotype and upland ecotype [3]. Lowland ecotypes are mostly tetraploid (2n = 4× = 36), and generally adapted to wet areas with milder winter temperatures, while upland ecotypes are mainly octaploid (2n = 8× = 72) or hexaploid (2n = 6× = 54), and thrive well in drier and colder areas. Lowland plants are usually taller and have longer and wider leaf blades, fewer tillers per plant, larger stem diameter and later in heading and flowering compared with upland plants [2,4,5].

Switchgrass germplasm collection and breeding for increasing biomass and conversion from cellulosic feedstock to ethanol have become a high priority [4,6,7]. In recent years, molecular markers have been extensively used to examine variation in switchgrass germplasm [5,8-13]. Switchgrass genetic linkage map has been established [14,15]. Three bacterial artificial chromosome (BAC) libraries have been generated [16,17]. Switchgrass expressed sequence tag (EST) database is being populated using traditional and RNA-Seq based technologies [18-21]. A switchgrass Affymetrix gene chip has been made available to the community through the DOE Bioenergy Science Center (BESC) co-operative efforts [21]. The procedure for switchgrass transformation has been established [22-24]. However, compared with rice, a well-studied monocot, studies on switchgrsass responses to biotic and abiotic stresses are limited [25-27].

Global warming predictions indicate that temperatures will increase another 2- 6°C by the end of this century [28]. High temperature can retard plant growth, development and yield, therefore, agriculture will be seriously affected by global warming in the future [29-31]. Based on the climate change observation, Behrman et al. [32] forecast that there will be substantial variation in switchgrass productivity within regions inside United States and over time. The southern United States, the main region of switchgrass production at present, is predicted to have the largest decrease in biomass in 2080–2090, due to the increased temperature and decreased precipitation [32]. Several studies reported the effect of high temperature on switchgrass focusing on physiology, composition and yields [27,33-36]. In the present study, we conducted transcriptome analysis using Affymetrix gene chips to elucidate the transcriptional changes in response to heat stress in Switchgrass Alamo, a lowland cultivar, extensively grown as biofuel feedstock. Comparative transcriptome analysis with other monocots identified a core set of 16 common heat stress responsive genes. The identified genes will provide rational candidates for germplasm screening to enhance switchgrass heat tolerance.

## Results and discussion

### Phenotypic responses to heat stress in switchgrass

**Figure 1:**
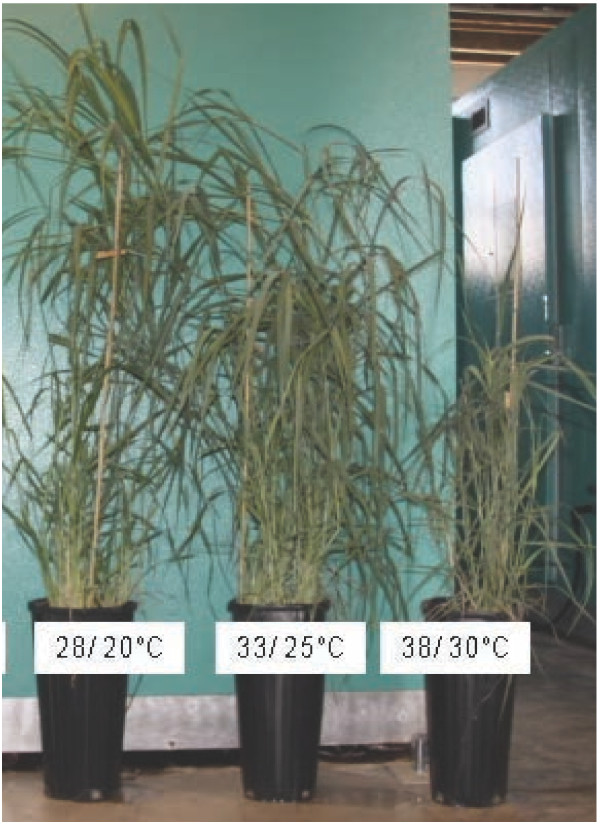
**Phenotypic responses to different temperature conditions in switchgrass Alamo.** Thirty days switchgrass seedlings grown at 28°/20°C (day/night) were subjected to high temperature treatment [33°/25°C and 38°/30°C (day/night)] for 50 days. Photograph was taken at the end of treatment. The optimal growth temperature is 28°/20°C (day/night).

Switchgrass plants grown under the optimal 28°/20°C (day/night) condition produced more foliage and were nearly twice the height of the plants that were under extreme heat stress at 38°/30°C (day/night). Plants that were subjected to a moderate heat stress treatment of 33°/25°C (day/night) showed only a slight reduction in the plant height (Figure 1). In a recent report on heat stress response in four switchgrass cultivars including Alamo, it was reported that stem elongation rate, leaf elongation rate and plant height were greatly impaired, ultimately lowering the growth and biomass of switchgrass cultivars at 38°/30°C (day/night) [27]. This reduction of total biomass by nearly 50% in response to elevated temperatures is a concern in the wake of the predicted increases in the global temperatures and particularly in the south-western USA, an ideal location for switchgrass production for biofuels. In fact, during the summer of 2011–2012 there were more than 100 days with more than 38°C temperature in the State of Oklahoma (http://www.mesonet.org).

### Transcriptome analysis in response to heat stress in switchgrass

A switchgrass affymetrix array containing 122,868 probesets corresponding to 110,208 unigene transcripts has been recently developed [21]. We used these gene chips to examine changes in switchgrass transcriptome following the heat stress treatment. The correlation between the two biological replicate experiments was 0.97. Using the arbitrary 2-fold cutoff about 4.2% of the probesets on the array was identified as heat-stress responsive. In several other plants about 5% of the transcriptome has been reported to be heat responsive [37-39], indicating that switchgrass heat stress transcriptional response is quantitively comparable to other plants.

Among the 5164 differentially expressed probesets in response to heat in switchgrass, 2076 probesets corresponding to 2002 switchgrass unigenes were induced, and 3088 probesets corresponding to 2809 switchgrass unigenes were repressed. In the heat transcriptome studies in maize, wheat, and rice, the number of induced genes was 3–6 times more than the number of repressed genes [38,40,41]. We surmise that the reason for this difference may be due to the differences in the heat stress treatment regimes. In the long-term heat stress imposed in our studies the observed transcript changes reflect the acclimative response. Whereas in the short duration heat stress in the other studies mentioned above, the transcriptional changes reflect the more active defense response.

**Figure 2:**
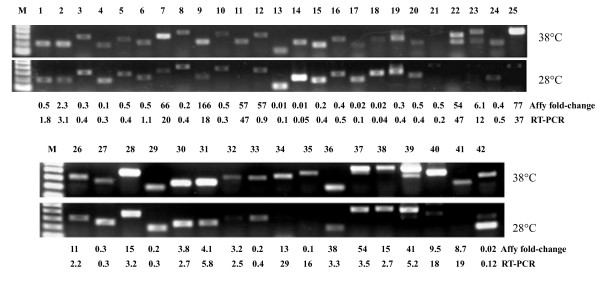
**Validation of heat stress regulated transcripts using RT-PCR.** Actin was used as the reference gene for normalization. Normalized fold change based on band intensities are indicated under gel picture. PviUT IDs 1. AP13CTG28453; 2. AlamCTG05039; 3. AP13CTG08489; 4. AP13CTG12099; 5. AP13CTG16627; 6. AP13CTG17205; 7. AP13CTG27166; 8. AP13ISTG54654; 9. KanlCTG02147; 10. KanlCTG03814; 11. AP13CTG14872; 12. AP13CTG15627; 13. AP13CTG20898; 14. AP13CTG22556; 15. AP13CTG24007; 16. AP13CTG29766; 17. AP13CTG31850; 18. AP13ISTG42000; 19. AP13ISTG49574; 20. AP13ISTG64132; 21. KanlCTG08905; 22. KanlCTG24811; 23. KanlCTG36075; 24. KanlCTG31732; 25. AP13ISTG61042; 26. AP13ISTG74729; 27. AP13ISTG68627; 28. AP13CTG27495; 29. AP13CTG27835; 30. KanlCTG24141; 31. KanlCTG22090; 32. AP13ISTG70341; 33. AP13CTG29083; 34. KanlSGLT55375; 35. KanlCTG47822; 36. KanlCTG09502; 37. AP13CTG13877; 38. KanlCTG00487; 39. AP13CTG27164; 40. AP13ISTG49055; 41. AP13ISTG32900; 42. AP13ISTG69974 (Sequences for these unigenes can be retrieved from http://switchgrassgenomics.noble.org). The corresponding rice orthologs, gene annotation, primer sequence and PCR cycles are listed in Additional file 1.

To validate the transcriptional pattern identified by microarray analysis, expression of 42 differentially expressed genes was analyzed using RT-PCR. The results showed that majority of the tested genes (38 genes) followed the expression pattern observed in the gene chip experiments (Figure 2, Additional file 1), validating the array results. In the few cases where there was a discrepancy it was observed that the hybridization intensities associated with those genes were weak for either the control or the heat stressed sample.

### Gene ontology analysis of switchgrass heat responsive transcripts

To investigate the biological significance of the genes regulated by heat stress in switchgrass it is important to have the gene ontology (GO) descriptions i.e., detailed annotations of gene function, biological process it is involved, and cellular location of the gene product. Since switchgrass genes have not been well annotated yet, rice orthologs of the differently regulated transcripts were identified. The best rice transcripts matching the switchgrass probesets (E-value > 1e10^-5^ and with at least 100 HSPs) were retrieved from the switchgrass genomics database maintained by the Samuel Roberts Noble Foundation (http://switchgrassgenomics.noble.org). Rice orthologs that showed 98-100% homology with the switchgrass probesets was identified for 4062 (78.7%) heat stress responsive switchgrass probesets. Among these, 1158 unique rice orthologs, representing 1478 switchgrass probesets were up-regulated, and 1857 unique rice orthologs, representing 2584 switchgrass probesets were down-regulated by heat stress. These unique rice orthologs were subjected to singular enrichment analysis (SEA) in agriGO to identify enriched GOs [42]. SEA is designed to identify enriched GO terms in a list of microarray probe sets or gene identifiers. Finding enriched GO terms corresponds to finding enriched biological facts, and term enrichment level is judged by comparing query list to a background population from which the query list is derived. In this study the background query list comprised of 54,971 annotated rice genes from the MSU 6.1 version (http://rice.plantbiology.msu.edu/).

**Figure 3:**
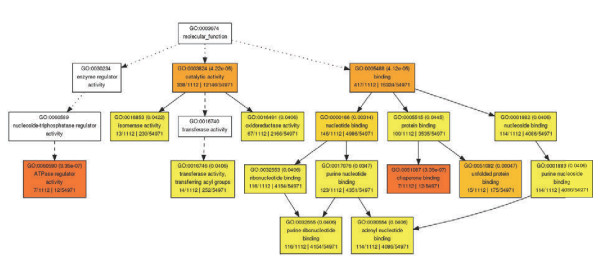
**Gene Ontology (GO) analysis of switchgrass heat-stress induced genes using agriGO.** Transcripts with more than 2-fold expression changes under heat stress compared with optimal temperature were designated as heat stress regulated gene. Each box shows the GO term number, the p-value in parenthesis, GO term. The first pair of numerals represents the number of genes in the input list associated with that GO term and the number of genes in the input list. The second pair of numerals represents the number of genes associated with the particular GO term in the rice database and the total number of rice genes with GO annotations in the rice database. The box colors indicates levels of statistical significance with yellow = 0.05; orange = e-05 and red = e-09.

Among the heat stress induced transcripts, GOs associated with ATPase regulator, protein folding, chaperone binding and catalytic activity were significant (Figure 3 and Additional file 2). Heat stress affects the stability of various proteins, membranes, RNA species and cytoskeleton structures. In order to counter the imbalance, survive and continue grow at a higher temperature, plants have to reprogram their transcriptome, proteome, metabolome and lipidome by altering the transcripts, proteins, metabolites and lipids [43-45]. It is well known Heat shock proteins (HSPs) and other chaperones are induced by various stresses. They play an important role in protein-protein interactions such as folding, assisting in proper protein conformation, stabilizing partially unfolded proteins and prevention of unwanted protein aggregation. The induction of HSPs expression is one of the common heat responsive mechanisms in all organisms [46-50]. Thus the enrichment of the GO associated with ATPase regulator, protein folding and chaperone binding in switchgrass is not surprising. There were 50 heat shock proteins and other chaperones that were strongly induced by heat stress in switchgrass, about 2/3 of these induced genes were also observed in rice panicle (Additional file 3). Since a large number of proteins will be misfolded under heat stress, there is a need for these chaperones to assist in the repair and/or salvage process. This is an energy demanding activity and apart from ATP requires the assistance of nucleotide exchange factor proteins such as GrpE [51]. Upregulation of the ATP synthase subunit along with three different GrpE genes in switchgrass is consistent with the findings in other plant species [38,40,41]. A switchgrass heat shock protein (ortholog of Os04g01740) was induced more than 66-fold by heat stress, and this pattern was further validated by RT-PCR (Figure 2). This gene was also induced 102 times in rice panicle under heat stress [41]. Other common heat stress response proteins included DnaK proteins (Os01g62290, Os03g11910 and Os03g16920), DnaJ protein (Os05g48810), and heat shock protein (Os02g52150, Os05g44340 and Os01g04370) (Additional file 3).

Under the GO category of catalytic activity, isomerase, transferase and oxidoreductase activities were enriched in response to heat stress in switchgrass (Additional file 2). The GO for oxido-reductase activity formed the largest group with 67 genes. Many of the genes in this group were associated with oxidative stress that is caused due to excess accumulation of ROS and/or due to insufficient antioxidant defenses [45,52-55]. A superoxide dismutase (SOD) (Os08g44770), two different amine oxidases and an alcohol oxidase were identified and could be involved in the generation of ROS during heat stress in switchgrass [56]. Six different peroxidases were induced, among them were cytosolic, stromal and peroxisomal ascorbate peroxidase and glutathione peroxidase. Interestingly, lack of chloroplastic ascorbate peroxidase resulted in enhanced heat stress tolerance in Arabidopsis plants [57]. This suggests that chloroplastic ROS, may play different role in switchgrass heat stress response. On the same lines, induction of thylakoid APX may be an important factor leading to heat sensitivity in Alamo plants. Cytosolic ascorbate peroxidases are important for protecting the organelles especially chloroplast ROS-scavenging system in Arabidopsis and lack of this enzyme can lead to increased protein oxidation [58]. The induction of two different peptide methionine sulfoxide reductase, a well-known enzyme vital for repairing oxidatively damaged proteins especially photosynthetic antennae [59], implies a severe oxidative stress due to high temperature in switchgrass.

Eleven different cytochrome P450 genes were identified in response to heat stress in switchgrass. Upregulation of two genes for CYP71A1 suggest the increased synthesis of indole alkaloids such as secologanin [60]. Induction of three different peroxiredoxins and a thioredoxin in switchgrass is again indicative of the oxidative stress induced by high temperature. Peroxiredoxins are important for reducing hydrogen peroxide and alkyl-hydroperoxides [61] and has been reported in response to heat stress in other plants [62]. In Arabidopsis, a thioredoxin was shown to form low to oligomeric protein structures and also high molecular weight complexes in response to heat stress [63]. Furthermore, the low weight structures showed disulfide reductase activity while the higher complexes exhibited chaperone function [63]. Interestingly, several targets of cytosolic thioredoxin [64], such as ascorbate peroxidase, malate dehydrogenase, glyceroldehyde-3-phosphate dehydrogenase, alcohol dehydrogenase were up regulated in response to heat stress in switchgrass. Further characterization of the switchgrass thioredoxin will provide a better understanding for the role of this protein in the heat stress response. A switchgrass DUF538 domain containing protein (ortholog of Os01g11240) expressed at very low level under normal condition was strongly induced by heat stress (56.7-fold). The function of DUF538 protein is unknown, however it has been proposed as putative candidate for the common stress related proteins in the plant system [65]. Exogenously applying maltose-binding fusion protein (MBP-DUF538) on the leaves of tobacco can elevate activities of redox enzymes including catalase, peroxidase, polyphenol oxidase and phenyalanine ammonia lyase [66]. We speculate that the switchgrass DUF538 protein is involved in ROS detoxification during heat stress.

The GO for the unfolded protein response consisted of 15 genes and six of them were annotated as T-complex proteins. The first member of this complex was identified in the hyperthermophilic archeon, *Pyrodictium occultum*, in response to heat stress and was shown to have ATPase activity [67]. Detailed structural analysis revealed that this enzyme is a complex of eight subunits and the fact that it is heat-inducible led to the name thermosome [68]. Careful sequence analysis of these switchgrass T-complex genes (data not shown) indicated that they were part of the group II chaperonin or the CCT complex [69].

Just as proteins become misfolded during heat stress, it is conceivable that the secondary structures of mRNAs can be disrupted in response to heat stress. We identified five different DEAD box RNA helicases that were up regulated in response to heat stress in switchgrass. Several studies on DEAD box RNA helicases in response to cold and salinity stress has been previously reported [70-74]. A rice DEAD box RNA helicase, OSABP, was strongly repressed in response to heat and cold stress [75]. A detailed analysis of the switchgrass DEAD box RNA helicases induced in response to heat merits further attention as these proteins may function as RNA chaperones.

Heat stress induces inward calcium flux, the increased calcium ion (Ca^2+^) level can in turn regulate multiple signaling pathway in plants [76]. The inward flux of calcium can activate several calcium-dependent protein kinases (CDPKs), which can, in turn, activate multiple mitogen-activated protein kinases (MAPKs) [77]. A calcium-transporting ATPase (Os04g51610), a calcium-dependent protein kinase CPK1 adapter protein (Os06g50146), calcineurin B (Os01g39770), and four calmodulin dependent protein kinases were induced in switchgrass by heat stress. In Arabidopsis, calmodulin AtCaM3 is required for heat stress signaling and is involved in the activation of WRKY and HSF transcription factors [78-81]. Similarly, the WRKY transcription factor (Os03g55164) and four HSFs associated with MAPK signaling were induced by heat stress in switchgrass. Surprisingly, a calreticulin precursor (Os07g14270) is the highest induced gene (166.4-fold) and was validated by RT-PCR (Figure 2 and Additional file 1). Calreticulin (CRT) is a key Ca^2+^-binding protein mainly resident in the endoplasmic reticulum in plants. CRT plays important roles in a variety of cellular processes including Ca2^+^ signaling, protein folding and as a key alleviator of endoplasmic reticulum stress [82,83]. CRT mRNA and protein are upregulated in response to cold stress, salt stress and exogenous phytohormones [83] and this study revealed that CRT is also induced in the heat stress response.

**Figure 4:**
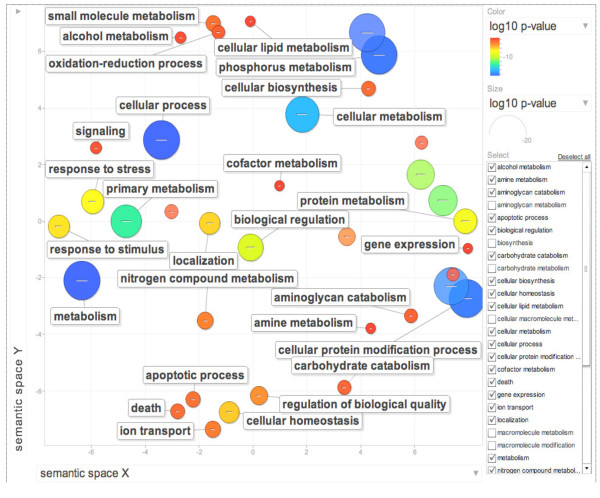
**Gene Ontology (GO) analysis of switchgrass heat stress repressed genes using REVIGO.** The scatter plot shows the cluster representatives (terms remaining after reducing redundancy) in a two-dimensional space derived by applying multi-dimensional scaling to a matrix of GO terms semantic similarities. Bubble color indicates the p-value for the false discovery rates derived from the AgriGO analysis. The circle size represents the frequency of the GO term in the uniprot database (more general terms are represented by larger size bubbles).

In proportion with the larger number of repressed genes in response to heat stress in switchgrass, more enriched GO categories were identified (Additional file 4 and Additional file 5). In order to reduce the number of GO terms, enriched GO categories with false discovery rates (FDR) < 0.05 from AgriGO analysis were submitted to the REVIGO (REduce and Visualize GO) program [84]. Using the Uniprot database as background and the default semantic similarity measure (Simrel), this analysis clearly showed that biological processes associated with metabolism, cellular homeostasis, cell death, regulation of transcription and transporters were significantly over-represented among the genes repressed by heat stress in switchgrass (Figure 4).

All the genes involved in glycolysis were strongly repressed by heat stress in switchgrass. The strong repression of the genes involved in glucose metabolism strongly supports the significant reduction reported in the cellulose and hemicellulose levels in response to heat stress in switchgrass cultivars [27]. The down regulation of metabolism in general, nitrogen and phosphorus metabolism in particular will have pronounced impact on vegetative growth (Figure 1). This is also supported by the observation that the dry shoot biomass in switchgrass cultivars was reduced by nearly 66% in response to heat stress [39]. These studies clearly demonstrate that under higher temperatures, genes associated with carbon fixation is down regulated, leading to reduced biomass. The reduction in the growth and development is probably brought into effect by the strong repression of a gamut of transcription factor families including 19 WRKYs, 13 NAMs (No Apical Meristem), 11 Myb TFs, nine AP2 domain containing TFs, five AUX/IAA type TFs (Additional file 6). Based on the predictions of higher temperatures in areas where switchgrass will be used as the main bioenergy crop our transcriptome studies indicate significant repression of carbon fixation processes that ultimately will have a negative impact on cellulosic bioethanol production.

### Comparative transcriptome analysis of heat stress response in monocots

In order to identify the unique responses and commonalities in response to heat stress among monocots we identified three other transcriptome studies in rice [41], wheat [38] and maize [40]. We examined transcriptome profiles from these studies using publicly available datasets. The switchgrass heat response transcriptome had the largest number of unique genes (2272). In contrast to the short-term heat stress treatments (few hours), long-term heat treatments in switchgrass (for up to 50 days) may be providing a totally different snap shot of the transcriptome. Among unique differentially expressed genes, 1462 were down regulated and 886 genes were up. Among the enriched GOs of repressed switchgrass-specific genes, redox homeostasis, regulation of transcription, transport and ubiquitination was particularly interesting since these were up regulated in the short-term heat treatment studies in rice [41]. In response to 24-h heat treatment in wheat, (considered as long-term heat stress), two different NADPH oxidases were induced while these genes were repressed in a short-term one hour treatment [38]. In contrast to short-term stress, we speculate that the long-term heat regime lead to excess oxidative stress that causes a redox imbalance. Similarly, short-term heat response invokes a sleuth of transcription factors in wheat and maize [38,40], while the long-term heat stress in switchgrass leads to repression of transcription factors that are associated with growth and development. This suggests that key processes involved in signaling and/or adaptation based on transcriptome are very different for short and long term heat stress treatments. However, it is important to realize that many of these genes exists as gene families and specific gene family members may be specific for certain stress and/or developmental stage as has been shown in maize and wheat [38,40]. GO enrichment analysis using the up regulated gene identifiers uniquely responsive to heat stress in switchgrass did not reveal any new categories at the level 3 terms i.e. two levels more specific than the top-level, molecular function or biological process.

**Figure 5:**
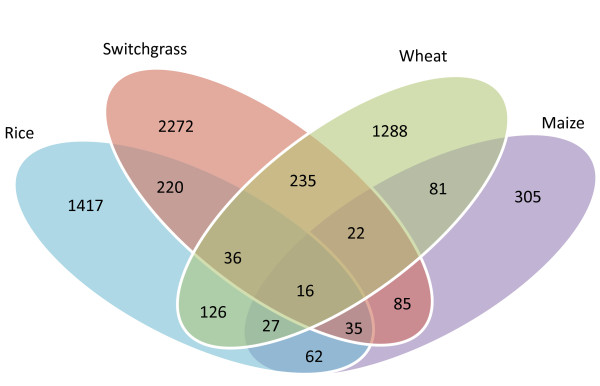
**Comparison of heat stress transcriptome of switchgrass, rice, wheat and maize.** The heat stress transcriptome data for rice was from reference 38, for wheat from reference 40 and for maize from reference 41. Rice orthologs for the maize and wheat heat responsive genes were identified using the microarray platform translator tool in Plexdb. Overlapping and unique gene identifiers were determined using Microsoft Excel.

Sixteen genes were identified in all the four monocots (Figure 5 & Table 1) despite the fact that the heat stress treatment conditions and the plant growth stages used in these studies were extremely different. Among them, only two genes were down-regulated (Table 1). Interestingly, the expression patterns of these 16 genes are very similar in the four different plant species (Table 1). The significant GO category of the common up-regulated genes is related with protein folding and unfolding, a common biochemical response to short-term and long-term heat stress.

**Table 1:** Sixteen common heat responsive genes in switchgrass, rice, maize and wheat

**PviUT ID annotation**	**Pv**	**Rice**					**Zm**	**Wheat**							
		**20 min**	**60 min**	**2 h**	**4 h**	**8 h**		**CS 1 sh**	**CS 1 h**	**CS 24 sh**	**CS 24 h**	**TAM 1 sh**	**TAM 1 h**	**TAM 24 sh**	**TAM 24 h**
AP13CTG24376 universal stress protein	2.5	2.7	6.8	7.9	2.6	1.9	7.2	13.2	28.4	4.5	5.4	13.1	23.1	2.2	2.7
KanlCTG39278 DnaK family protein	3.4	3.2	3.2	2.9	2.0	1.8	4.1	30.7	39.4	12.8	19.6	33.2	40.3	12.8	17.3
AP13ISTG73695 glycosyl hydrolases family 17	0.1	2.4	0.4	6.5	7.2	11.5	0.5	0.9	0.8	1.7	1.3	0.3	0.2	0.2	0.1
KanlSGLT49533 activator of 90 kDa HSP ATPase homolog	2.2	8.0	7.1	4.8	2.8	1.8	5.0	6.1	11.1	2.5	3.3	5.9	12.7	2.0	2.6
AP13CTG04492 chaperone protein clpB 1	2.2	20.5	15.7	6.8	2.8	1.5	3.3	9.5	74.4	2.1	2.4	37.5	165	3.3	4.0
KanlCTG05390 heat shock 22 kDa protein, mitochondrial precursor	4.1	180	65.9	38	19	23.3	3.4	172	268	11.9	11.5	106	132	6.9	8.6
AP13CTG00593 T-complex protein	2.2	3.2	3.8	2.0	1.2	0.7	2.4	4.2	11.3	2.1	2.8	6.7	17.4	3.6	3.3
KanlCTG37554 Putative transposon	0.5	0.9	0.4	0.3	0.2	0.2	0.5	0.3	0.1	0.6	0.5	0.3	0.2	0.8	0.7
AP13CTG06779 T-complex protein	2.4	5.7	6.8	3.4	3.5	2.2	2.5	8.1	29.3	3.1	3.4	12.5	53.3	3.3	3.0
AP13CTG59854 expressed protein	2.1	3.7	4.6	2.2	1.2	0.9	2.5	5.9	6.7	5.0	4.7	4.4	5.7	5.0	4.7
AP13CTG14658 chaperonin	3.6	5.3	4.2	2.9	2.0	1.4	4.3	14.1	13.0	10.4	11.2	14.7	14.8	9.3	10.2
AP13ISTG34202 activator of 90 kDa HSP ATPase homolog	2.4	6.0	4.9	2.9	1.8	1.3	3.4	5.2	13.4	2.7	2.9	6.1	14.7	2.1	2.3
AP13CTG25439 peptidyl-prolyl isomerase	3.4	21.9	23.4	9.7	4.2	1.8	3.9	15.1	21.2	6.9	5.9	11.6	15.6	5.0	5.1
AlamCTG07708 co-chaperone GrpE protein	2.1	4.1	5.7	4.7	4.8	3.6	2.3	4.1	3.7	3.1	3.2	4.7	4.2	2.8	2.6
AP13CTG01830 T-complex protein	4.5	3.6	3.3	3.0	2.2	1.5	5.8	1.3	0.7	2.2	2.0	1.3	0.7	2.3	2.3
AP13CTG30798 HIT zinc finger domain protein	3.2	8.4	8.5	6.3	4.4	2.8	2.4	1.8	1.5	2.4	2.4	1.9	1.8	2.0	2.2

Pv is *Panicum virgatum*; Zm is *Zea mays*. Two wheat genotypes, heat susceptible “Chinese Spring” (CS) and heat tolerant “TAM107” were used and the data is derived from [38]. Maize data was retrieved from reference [40] and rice data were from reference [41].

Members of 'Activator of Hsp90 ATPase’ bind to the molecular chaperone HSP82 and stimulate its ATPase activity [85]. Small heat shock protein, sHsps, are small stress induced proteins with monomeric masses between 12–43 kDa, are believed to be ATP-independent chaperones that prevent aggregation and are important in refolding in combination with other Hsps [86]. GroEL_like type I chaperonin are involved in productive folding of proteins and in plants are called as cpn60 [87]. With the aid of co-chaperonin GroES, GroEL encapsulates non-native substrate proteins inside the cavity of the GroEL-ES complex and promotes folding by using energy derived from ATP hydrolysis. Chaperonin 10 kDa subunit (cpn10 or GroES) cooperates with chaperonin 60 (cpn60 or GroEL), an ATPase, to assist the folding and assembly of proteins and is found in cytosol, as well as in the matrix of mitochondria and chloroplasts. It forms heptameric rings with a dome-like structure, forming a lid to the large cavity of the tetradecameric cpn60 cylinder and thereby tightly regulating release and binding of proteins to the cpn60 surface. The 60 kDa chaperonin alpha subunit is a part of the T-complex proteins important in the unfolded protein response of cytoskeletal proteins-actin and tubulins and also other proteins [69]. Aha1 is one of several co-chaperones, which regulate the dimeric chaperone Hsp90. Hsp90, Aha1, and other accessory proteins interact in a chaperone cycle driven by ATP binding and hydrolysis. Aha1 promotes dimerization of the N-terminal domains of Hsp90, and stimulates its low intrinsic ATPase activity [88]. Aha1 may regulate the dwell time of Hsp90 with client proteins. Aha1 may act as either a negative or positive regulator of chaperone-dependent activation, depending on the client protein [89]. GrpE is the adenine nucleotide exchange factor of DnaK (Hsp70)-type ATPases and is important for thermo-tolerance to chronic heat stress in plants [51]. The GrpE dimer binds to the ATPase domain of Hsp70 catalyzing the dissociation of ADP, which enables rebinding of ATP, one step in the Hsp70 reaction cycle in protein folding and can direct incompetent “client” proteins towards degradation [90]. ATP-dependent Clp protease, ClpB, has been described as HSP101 in plants and is indispensable for basal thermotolerance and negatively impacts root growth [91] though in the absence of stress, this protein is dispensable for normal growth and development.

The universal stress protein (Usp) is a small cytoplasmic protein whose expression is enhanced when the cell is exposed to stress agents. Usp enhances the rate of cell survival during prolonged exposure to such conditions, and may provide a general “stress endurance” activity [92]. FKBP-type peptidyl-prolyl cis-trans isomerase with a Tetratricopeptide repeat domain is involved in chaperone, cell-cycle, transcription, and protein transport complexes. We speculate that in response to heat stress it most likely behaves as a chaperone. Identification of a gene similar to beta-1,3-glucanase suggests that similar to the biotic stresses, modification of cell walls is an important component of the heat stress response. L-Asparaginase type 2-like enzymes are important for nitrogen remobilization and seed production [93]. Asparaginase has been shown to be important for low temperature response in soybeans [94]. Asparaginase as a common component in the heat stress response in plants may be important for remobilizing the scanty nitrogen reserves for ensuring seed development.

## Conclusions

The present study identified significantly altered transcripts in switchgrass under chronic heat stress. Using a comparative transcriptome analysis we identified 16 common genes in the heat stress response in plants. Since these genes are expressed both under short and long-term heat stress treatments, they are ideal biomarkers for screening germplasm for thermo-tolerance. This will be valuable for developing new plant types that can adapt and thrive well under high temperatures that are predicted for the future.

## Methods

### Plant growth and treatment

Switchgrass (*Panicum virgatum* L.) Alamo cultivar seeds were sown in pots (0.2 meter diameter × 0.45 m tall) filled with pure, fine sand soil and grown in growth chambers (Conviron Ltd., Winnipeg, Canada) at 28°/20°C (day/night) with a photoperiod of 14 h/10 h (day/night). After germination, switchgrass seedlings were thinned to four plants per pot. Thirty days after sowing, switchgrass seedlings were subjected to heat stress treatment of 38°/30°C (day/night) for 50 days. Leaf samples from plants growing under control condition (28°/20°C) and heat stress (38°/30°C) were harvested and snap frozen in liquid nitrogen for RNA isolation at the end of treatment.

### RNA isolation and switchgrass affymetrix genechip hybridizations

Total RNA from switchgrass leaves was isolated using RNEasy Plant Isolation kits (Qiagen Valencia, CA, USA). The quality of the RNA was tested using the BioAnalyzer (Agilent, Palo Alto, CA, USA). About 10 μg RNA was used for probe labeling according to procedure of GeneChip 3’ IVT Express Kit (Affymetrix, Santa Clara, CA). Labeled probe was hybridized to Affymetrix switchgrass cDNA chip containing more than 120,000 probe sets. Hybridizations were done in duplicates using RNA prepared from two biological replicate samples.

### Microarray data analysis

Microarray data normalization was conducted by robust multi-array average (RMA) provided with Expression Console [95]. Probesets showing a normalized transcript level of more than 28 were selected for further analysis. The reason for this cut-off is that values obtained from probe sets with 'absent’ values, as determined by the Affymetrix software, consistently exhibited log 2 normal distribution with an upper boundary below 5 (log_2_28 = 4.8). Furthermore, the 20 negative controls probe sets detected no transcripts when hybridized to switchgrass RNA using this threshold. Probesets showing more than two-fold change (heat stress/control) in expression were considered as differentially regulated genes.

### Analysis of enriched gene ontologies

The best rice transcripts matching the switchgrass probesets (E-value > 1×10^-5^ and >100 HSPs) were retrieved from the switchgrass genomics database maintained by the Samuel Roberts Noble Foundation (http://switchgrassgenomics.noble.org). Rice orthologs of switchgrass genes were input for Gene ontology analysis using agriGO (http://bioinfo.cau.edu.cn/agriGO/)[42] and REVIGO (http://revigo.irb.hr/) [84] software.

### Gene expression validation

One microgram total RNA was used for cDNA synthesis with superscript reverse transcriptase II (Invitrogen). A 1:10 dilution of cDNA was used for PCR amplification. Forty-two differentially expressed genes were selected for RT- PCR analysis. Amplification of actin gene was used as the reference for normalization. The gene list, primer sequence, and corresponding rice orthologs are listed in Additional file 1.

### Comparative transcriptomics

A detailed analysis of the rice heat responsive transcriptome was published recently [41]. In the rice study 3364 heat responsive probesets, represented 3213 rice genes, of which 2451 were identified as unique loci. These 2451 unique differentially expressed genes were used for comparative analysis with the genes identified in switchgrass analysis. Nearly 6560 differentially expressed probesets were reported in response to heat stress treatment in wheat [38]. Rice orthologs of the wheat probesets were retrieved using the 'microarray platform translator’ tool in PLEXdb [96]. Using this tool, 5288 rice probesets were identified while for 1272 probesets there were no orthologs using the default BLAST parameters. The rice affymetrix probe identifiers were then fed into the model genome interrogator tool in the PLEXdb to retrieve the corresponding rice locus identifiers. This analysis yielded 1831 unique rice loci. A heat transcriptome analysis in maize using the long oligo array reported 1081 differentially expressed genes [40]. Using the PLEXdb microarray platform translator tool 887 corresponding rice orthologs were retrieved and this corresponded to 633 unique rice loci. A four-way comparison of the heat responsive transcriptomes of rice, wheat, maize and switchgrass was undertaken.

## Availability of supporting data

The microarray data sets supporting the results of this article are available in the ArrayExpress repository under the accession number E-MTAB-1897.

## Authors’ contributions

RM received grant support. RM, YW and YL designed the experiment. VGK grew switchgrass plants, performed the heat stress treatments and collected the tissue samples. YT performed the affymetrix genechip hybridizations, conducted the preliminary data analysis and assisted in submitting the microarray data to ArrayExpress. YL and YW validated gene expression. YL and RM analyzed the array results and prepared the manuscript. All authors reviewed and approved the final manuscript.

## Supplementary Material

Additional file 1 RT-PCR validation of differentially expressed transcripts identified by microarray. Click here for file

Additional file 2 Enriched GO categories of the heat stress induced transcripts in switchgrass. Click here for file

Additional file 3 Heat shock proteins and other chaperones induced in switchgrass by heat stress. Click here for file

Additional file 4 GO analysis of switchgrass heat-repressed genes using agriGO. Click here for file

Additional file 5 Enriched GO categories of the heat stress repressed transcripts in switchgrass. Click here for file

Additional file 6 Transcription factors down regulated by heat stress in switchgrass. Click here for file
