# Supplementary figures and images for: Transcriptome analysis of heat stress response in switchgrass (Panicum virgatum L.)
Source: BMC Plant Biol. 2013 Oct 6;13:153. doi: 10.1186/1471-2229-13-153 (PMC3851271; doi:10.1186/1471-2229-13-153)

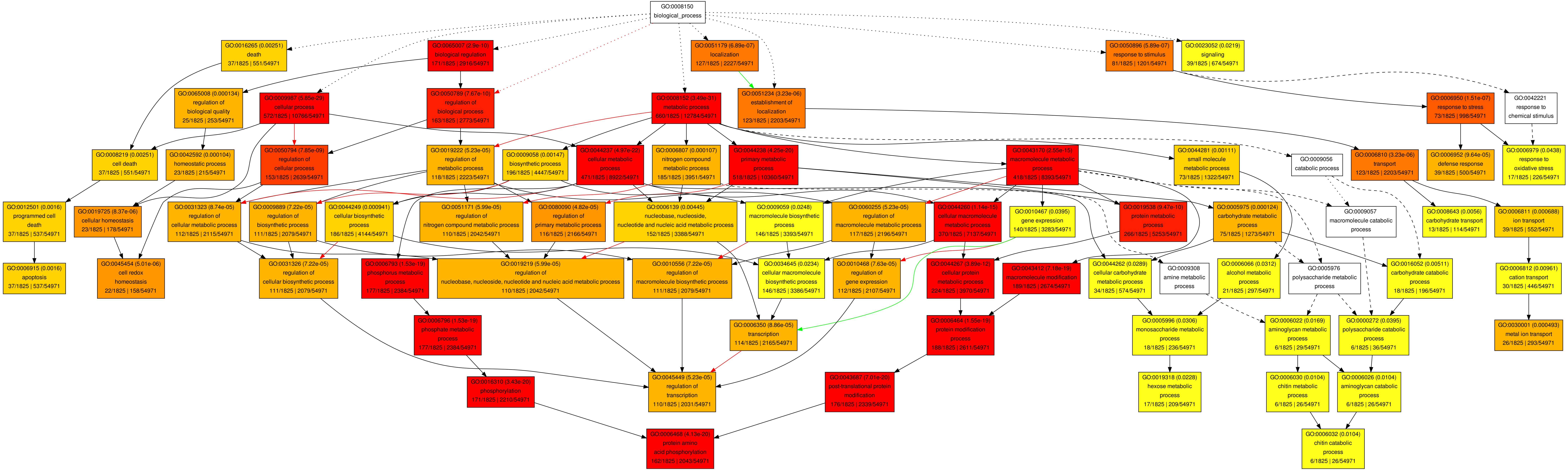

Supplement: Additional file 4 — GO analysis of switchgrass heat-repressed genes using agriGO. [file 1471-2229-13-153-S4.pdf]
